# Recent Progress on Modified Gum Katira Polysaccharides and Their Various Potential Applications

**DOI:** 10.3390/polym14173648

**Published:** 2022-09-02

**Authors:** Mahendra Singh, Chaitany Jayprakash Raorane, Alka, Divya Shastri, Vinit Raj, Seong-Cheol Kim, Minkal Tuteja

**Affiliations:** 1 Molecular Genetics Laboratory, Department of Biotechnology, Institute of Biotechnology, College of Life and Applied Sciences, Yeungnam University, Gyeongsan 38541, Korea; 2 School of Chemical Engineering, Yeungnam University, Gyeongsan 38541, Korea; 3 Sunder Deep Group of Institutions, Sunder Deep Pharmacy College, Ghaziabad 201002, India; 4 School of Pharmacy, Yeungnam University, Gyeongsan 38541, Korea; 5 Gurugram Global College of Pharmacy, 5 KM Milestone, Kheda Khurampur, Farrukhnagar-Haily Mandi Road, Gurgaon 122506, India

**Keywords:** gum katira, modifications, characterization, grafting, cross-linking applications

## Abstract

Gum katira polysaccharide is biocompatible and non-toxic, and has antioxidant, anti-microbial, and immunomodulatory properties. It is a natural polysaccharide and exudate derived from the stem bark of *Cochlospermum reliogosum* Linn. Additionally, it has many traditional medicinal uses as a sedative and for the treatment of jaundice, gonorrhea, syphilis, and stomach ailments. This article provides an overview of gum katira, including its extraction, separation, purification, and physiochemical properties and details of its characterization and pharmacognostic features. This paper takes an in-depth look at the synthetic methods used to modify gum katira, such as carboxymethylation and grafting triggered by free radicals. Furthermore, this review provides an overview of its industrial and phytopharmacological applications for drug delivery and heavy metal and dye removal, its biological activities, its use in food, and the potential use of gum katira derivatives and their industrial applications. We believe researchers will find this paper useful for developing techniques to modify gum katira polysaccharides to meet future demands.

## 1. Introduction

Recently, polysaccharides of natural origin and their derivatives have fascinated researchers due to their diverse applications, especially those requiring biocompatibility and biodegradability [1]. However, the short life spans and limited solubilities of polysaccharides restrict their use. Nonetheless, the functional groups and properties of these polysaccharides enable selective biochemical or chemical modifications of polymeric a backbone, which substantially broadens their applications and enables the tailoring of their bioactivities [2,3,4,5]. Polysaccharides can be categorized based on their plant of origin and the part of the plant harvested. For example, tamarind gum, locust bean gum, and guar gum are obtained from the seed [6,7]; pectin, cellulose, and hemicelluloses are cell polysaccharides [8]; and gum ghatti, acacia gum, and tragacanth gum are plant exudates [9]. Numerous modifications have been devised to develop new derivatives, and plant-based materials have a long history of use in drug delivery [10], tissue engineering [11,12], waste-water treatment [13], food packaging [14], and heavy metal removal [15].

Gum katira is a natural plant exudate obtained from the stem bark of *Cochlospermum religiosum* [16,17]. The tree produces gum from deeply furrowed bark when subjected to physical injury, stress, or fungal attack [18]. The gum contains D-galactose, L-rhamnose, and D-galacturonic acid in a molar ratio of 3:2:1 with trace amounts of ketohexose [19]. Gum katira and its derivatives exhibit specific characteristics, such as high levels of swelling in water, significant viscosity, excellent flow properties, and high water retention [17]. Importantly, gum katira is safe, non-toxic, and non-allergenic [20,21]. Formulations developed using gum katira and its derivatives have been reported to exhibit antimicrobial [22], anticancer, and anti-inflammatory activities [23], and they are also used as stabilizers, emulsifiers, and thickeners in the food industry [24].

Several means of modifying gum katira have been described in the literature, such as grafting [25], crosslinking [13,26], carboxymethylation [22], and sulfation [15,25], and these modifications can enhance its physicochemical properties, chemical stability, and mechanical characteristics. Over the past few years, gum katira has been used as a natural polysaccharide for the preparation of drug delivery formulations [23,27,28,29]. Furthermore, gum karaya hydrogels have been formulated to expand their applications for impurity removal and in the pharmaceutical and biomedical industries [1,25,26]. 

The values of grafting procedures are determined by their ability to provide simple and compact side chain refinement in grafting copolymers [30]. For polymeric graft modification, a variety of monomers are utilized, such as acrylamide, methyl methacrylate, acrylamides, N-acrylonitrile, tert-butylacrylamide, and N-polyvinylpyrrolidone [31,32,33]. Grafted copolymers have numerous applications for drug delivery, wastewater treatment, and the food and textile industries [34]. Microwave-aided modification is an alternative technique that allows rapid synthesis and is reproducible, straightforward, and environmentally compatible [35,36]. Furthermore, by adjusting exposure time, degrees of grafting can be regulated. On the other hand, gamma radiation, which modifies the polymeric backbone, is used to improve polymer mechanical properties. Grafting percentages increase with monomer content and radiation dose [37,38]. This approach is used to create functional micro- and nanospheres, hydrogels, interpenetrating networks [39], and hydrogels, and is relatively straightforward, clean, chemical initiator-free, and environmentally benign [40].

Over the last few years, considerable progress has been made in the development of gum katira and modified gum katira polysaccharides for different applications. This review article provides an overview of the pharmacognostic features of gum katira and its extraction, purification, and composition. In addition, this review describes the current situation regarding modification methods, applications of these variants in various fields, and the patent situation. The present review will be of interest to the scientific community and should aid the design and development of gum katira and its applications in pharmaceutical and other fields.

## 2. Pharmacognostic Study of *C. religiosum*

*C. religiosum* is a deciduous, small or medium-sized, soft-wooded tree and is found throughout India from Garhwal to the west sub-Himalayan territories to Bihar, Orissa, West-Bengal, and the Deccan peninsula. The tree is also known as the Golden silk cotton tree, Butter cup tree, or Yellow silk cotton tree, and is native to India, Thailand, and Burma [41]. Its flowers are used for offerings in temples and aesthetic purposes. Macromorphologically, the plant has simple, palmately lobed, alternate, distichous leaves with prominent parallel venation, acute apices, and crenate margins. The plant bark is smooth, fibrous, and ash-like in color, and its stems exudate an orange gum-like material. At the microscopic level, the leaves exhibit long filamentous unicellular uniseriate covering trichomes, a dorsiventral lamina, annulated lignified xylem vessel, anomocytic stomata, mucilaginous brown matter, and prisms of calcium oxalate and starch grains, while the bark exhibits lignified cork cells, bundles of lignified fibers, thick-walled lignified sclereids, elongated cellulosic medullary rays, rounded starch grains, rosettes, and tetragonal crystals of calcium oxalate. Inorganic elements in the leaves and bark include aluminum, calcium, sodium, chlorides, and iron, which were used to establish the quality standard for the crude drug of *C. religiosum* [16]. 

Parts of *C. religiosum* have been used to treat inflammation, tuberculosis, asthma, cough, jaundice, gonorrhea, and dysentery from ancient times [41,42,43,44,45,46]. Its dried flowers and leaves were used as laxatives, stimulants, antipyretics, and sedatives [47], while a paste prepared from stem bark was used as a plaster to treat bone fractures [48]. Furthermore, *C. religiosumgum* has a wide variety of veterinary uses [49,50,51,52,53,54,55], and the plant has been shown to possess antibacterial, antifungal, insecticidal, antioxidant and hepato protective activities [56,57]. In addition, the flowers, dried leaves, and latex of *C. religiosum* have been used to treat mouth ulcers and asthma [58], and powdered bark in water was used to treat jaundice [43]. 

The gum katira is obtained from *C. religiosum* Linn. (Family *Cochlospermaceae*) and has been used in foods and pharmaceutical preparations for thousands of years [19,59,60]. Gum katira has been used in the manufacture of cigars, ice-cream, and pastes, but more recently has been reported to have various pharmacological applications for the treatment of pharyngitis, diarrhea, gonorrhea, dysentery, trachoma, and syphilis [24]. The gum is also used in cosmetics to prevent skin diseases and soften skin [61]. Gum katira is acid resistant and not degraded in the stomach but is degraded by anaerobic microflora in the colon [62] and has been reported to be an appropriate carrier for colon-targeted drug delivery [28].

## 3. Extraction, Isolation, and Purification of Gum Katira

Isolation, extraction, and purification methods are important for obtaining good quality gum, which when collected is thermogenic, semi-transparent, pale, sweet in taste, a sedative in nature, and slightly soluble in water, though it swells in water to form a transparent mass. Purification is a crucial step since the gum contains various impurities, such as inorganic salts, low-molecular-weight proteins, non-polar materials, high-molecular-weight organic impurities, monosaccharides, and oligosaccharides as well as the desired polysaccharides [63]. Accordingly, the isolation and purification process must not damage the polysaccharides. Water or water-miscible solvents are used as the main solvents followed by alkaline water, and water- and alkali-soluble polysaccharides are separated according to their solubilities. Inorganic salts are removed using ion-exchange resins [64], but, at the commercial level, mixed beds of ion-exchange resins are employed. Impurities can also be removed by column chromatography, and polysaccharides can be purified by gel permeation chromatography [65]. Many polysaccharides can be separated and purified by centrifugation based on the relations between centrifugal force, molecular weight, and sedimentation rate [66,67]. Furthermore, ultrafiltration methods can also be used to purify polysaccharides based on the shape and size of polysaccharide molecules [68]. 

**Figure 1:**
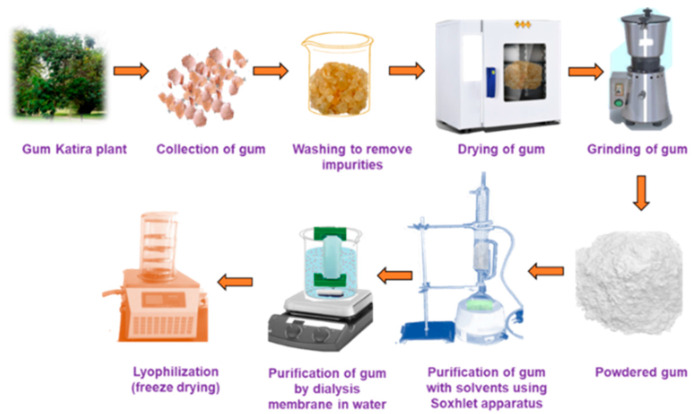
Schematic representation of gum katira collection and purification.

Briefly, after harvesting, gum katira is washed with methanol and dried. The dried gum is then powdered and subjected to sequential Soxhlet extraction using petroleum ether at 60–80 °C for 3 h, petroleum ether–benzene (1:1) for 72 h, and benzene-chloroform (1:1) for 72 h to obtain a white solid [25,60]. This solid is then dispersed in distilled water, dialyzed through a cellulose membrane to eliminate low-molecular-weight materials, centrifuged, and the filtrate is freeze dried. The pure polysaccharide is then obtained by Sepharose gel permeation chromatography. A schematic of the purification process is provided in Figure 1.

## 4. Structure Elucidation of Gum Katira

The phytochemical screening of *C. religiosum* Linn. indicates the presence of many constituents, such as alkaloids, glycosides, flavonoids, phenolic, saponins, steroids, coumarins, and leucoanthocyanidins [69,70], although isorhamnetin-3-glucosides (methylated quercetin) and myricetinare have also been isolated [70,71]. It has taken two decades (1950–1970) to understand and establish the structure of gum katira. Hirst and Dustan tried to determine the composition of gum katira and concluded that it contains equimolar proportions of rhamnose, D-galactose, and D-galacturonic acid with trace amounts of keto-hexose and that d-galactose, l-rhamnose, and d-galacturonic acid are present in a molar ratio of 3:2:1 [19]. Aspinall et al. reported the presence of 1→2, 4- linked d-galacturonic acid in the inner chain of a polysaccharide with residual neutral sugars [59]. 

**Figure 2:**
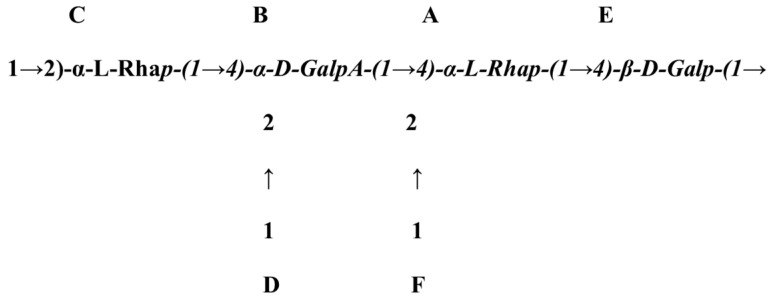
Representation of the proposed structure of gum katira polysaccharide Reprinted/adapted with permission from [60].

The structure of polysaccharides can be determined using chemical methods, such as methylation, acid hydrolysis, periodate oxidation, and Smith degradation, or spectroscopic methods, such as 1D NMR (^1^H, ^13^C, and DEPT-135) and 2D NMR (DQF-COSY, TOCSY, NOESY, ROESY, HSQC, and HMBC) [18]. Using these methods, researchers confirmed the structure of gum katira and concluded that the polysaccharide unit (Figure 2) consists of terminal L-rhamnopyranoside and (1→2,4)-linked L-rhamnopyranose, terminal D-galactopyranose, (1→4)-linked D-galactopyranose, (1→2)-linked L-rhamnopyranose, and amount of galacturonic acid (Figure 2) [60].

## 5. Physicochemical Properties of Gum Katira and Characterizations

Gum katira has been explored as an excipient and drug delivery carrier. Therefore, to establish its suitability as a pharmaceutical excipient, various physicochemical properties, such as particle size distribution, solubility, total microbial load, ash value, loss on drying, moisture content, acid volatile content, and rheo kinetics, must be determined using a variety of advanced analytical techniques. The physicochemical properties of gum katira are summarized in Table 1.

### 5.1. Fourier Transform Infrared (FTIR)

**Figure 3:**
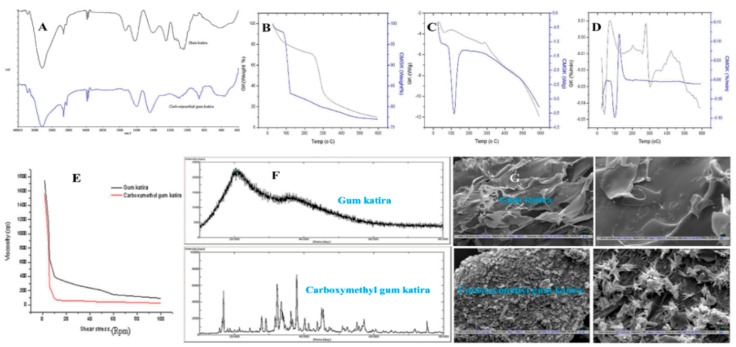
(**A**) FTIR, (**B**) TGA, (**C**) DSC, (**D**) DTG, (**E**) Viscosity, (**F**) XRD, and (**G**) SEM analysis of gum katira and carboxymethylated gum katira. Reprinted/adapted with permission from [22]).

The FTIR is used to identify functional groups in gum katira. Gum katira and carboxymethylated gum katira showed various characteristic peaks [22]. The FTIR spectra of gum katira and the carboxymethylated gum are shown in Figure 3A. Peak attributions for gum katira were as follows: The broad absorption at 3430 cm^−1^ was attributed to hydroxyl; the peak at 2947 cm^−1^ to alkane -CH stretch; the peaks at 1618 cm^−1^ and 1734 cm^−1^ to ketone and aldehyde stretch, respectively; peaks at 1254 and 1077 cm^−1^ to alcohol C-O-H and ether C-O-C stretch, respectively; and the peak at 1406 cm^−1^ to O-H bending vibration. A broad hydroxyl peak at 3330 cm^−1^ was previously assigned to the glucopyranose ring [17]. The carboxylic (-COOH) group produced a broad and intense -OH stretch absorption. Previously, C=C stretch was attributed to peaks at 1080 and 972 cm^−1^ [17,22]. For carboxymethylated gum katira, broad bands at 3432 cm^−1^ and 3771 cm^−1^ were assigned to alcohol -OH stretch, and peaks at 2926 and 2854 cm^−1^ to alkane C-H stretch. A peak at 1443 cm^−1^ not observed in the spectrum of gum katira was assigned to symmetric and asymmetric carboxylate anion (-COO-) stretch, and the peak at 1603 cm^−1^ to –CH_2_ scissoring vibration of the carboxymethyl group (Figure 3A).

### 5.2. Differential Scanning Calorimetry (DSC)

DSC determines the heat gain or loss resulting from chemical or physical changes in test samples as a function of temperature [73]. A sharp, symmetrical endotherm point is indicative of purity, while a broad asymmetric band indicates the presence of impurities [74]. An endothermic peak generally indicates a loss of water. The breakdown/melting of a crystalline solid requires a certain amount of energy and results in a sharp peak in its DSC thermogram [75]. Hence, this technique is applied to determine the existence of endo- and exothermic shifts on increasing temperature. Due to its accuracy and sensitivity, DSC is being broadly used to analyze polymer phase transitions [76]. The DSC curve of gum katira exhibited a broad endotherm at 63.20 °C, while that of carboxymethylated gum katira had a broad endotherm at 116.15 °C. This change in endotherm temperature confirmed the successful carboxymethylation of gum katira (Figure 3B) [22].

### 5.3. Thermogravimetric Analysis (TGA)

TGA is a simple, quantitative, and accurate technique used to investigate the thermal decomposition pattern of polymers. Outputted thermograms are plots of % mass (weight change) versus temperature. Thus, thermostable compounds exhibit no mass change at normal test temperatures [77]. TGA spectrograms of gummy exudates have enabled mechanisms of decomposition to be elucidated [78]. In one study, more than 50% of the gum had decomposed at 806.30 °C [17]. TGA results for gum katira are shown in Figure 3C and reveal thermal stability was enhanced by carboxymethylation [22].

### 5.4. Differential Thermal Gravimetry (DTG)

DTG requires that a test sample and an inert reference sample be subjected to identical thermal cycles. The results are out put as curves that provide information on changes in state, for example, crystallization, glass transitions, sublimation, or melting. DTG is used as a fingerprinting method in pharmaceutical research, the food and cement industries, mineralogical research, and environmental studies [78,79,80]. The DTG analysis of gum katira revealed four stages of degradation [22]. In the first stage, weight loss was attributed to loss of physisorbed water and the elimination of structural water. In the second stage, it was attributed to depolymerization and rupture of C-O and C-C bonds of the saccharide ring, which resulted in a weight loss of 30.78%, and in the third and fourth stages to degradation of polymeric chains. On the other hand, carboxymethyl gum katira showed two stages of breakdown. The first was attributed to water loss (a weight loss of 17.50%), and the second to depolymerization and polymer chain degradation (a weight loss of 5.56%). Furthermore, at 600 °C, gum katira and carboxymethyl gum katira had residual masses of 9.98% and 76.94%, respectively, which confirmed the better thermal stability of gum katira (Figure 3D).

### 5.5. Rheological Properties

The rheological behaviors of drilling fluids play a significant role in eliminating borehole problems [81]. The effects of gum katira and xanthan gum on the rheological behaviors of bentonite-water-based drilling fluid were investigated by considering various parameters like gum types, test temperature, and gum concentration. The apparent viscosities and yield points of base fluids increased on increasing the concentration of gum katira, and the yield point and plastic viscosity ratio depended on gum katira concentration and temperature (Figure 3E).

### 5.6. Powder X-ray Diffraction

The shapes of solids are dependent on 3D arrangements of molecules, atoms, or ions, and these determine, for example, whether the resulting solid is crystalline or amorphous (Figure 3F) [82]. Crystalline substances have sharp melting points, well-defined edges and faces, and diffract X-rays, whereas amorphous solids have irregular surfaces and do not produce X-ray diffraction patterns. The X-ray diffraction pattern of gum katira was amorphous with no strong peaks, whereas the diffractogram of carboxymethylated gum katira had diffraction peaks at 16.87°, 28.14°, 29.54°, 32.34°, 33.50°, 36.30°, 37.38°, 40.42°, 41.14°, 43.74°, 45.05°, 54.93°, 57.33°, and 75.20° at 2θ [22]. The amorphous form of gum katira, which probably contributes to its swelling and hydration properties, was well reflected by its X-ray diffraction pattern, whereas the diffraction pattern of carboxymethylated confirmed its crystalline nature by exhibiting characteristic sharp, high-intensity peaks [22]. Similar studies on the carboxymethylation of other polysaccharide gums have also reported that carboxymethylation increased crystallinities [83,84].

### 5.7. Scanning Electron Microscopy (SEM)

Shape and surface morphology is generally determined by SEM. As demonstrated in Figure 3G, SEM pictures of gum katira revealed smooth-surfaced thin flakes, whereas photomicrographs of carboxymethylated gum katira revealed the presence of polyhedral particles with rough, sharp-edged surfaces [22].

### 5.8. Gum Katira Toxicity Studies

Toxicity studies in vitro and in animal models are essential for determining the safety profiles of materials before clinical trials. These studies should be conducted in compliance with the guidelines issued by the Organization for Economic Cooperation and Development (OECD) and relevant institutional ethics committees when appropriate. Researchers have reported gum katira is non-toxic at doses ≤ 2 g/kg body weight daily for 14 to 30 days in animal and that no behavioral alterations were detected during the first 4 h and no mortality was observed after 24 h when animals were administered the dose [20,21]. In a sub-acute toxicity investigation, the hematological analysis revealed no significant differences between treated and non-treated control animal groups [20,72]. Furthermore, when administered orally to rats, hematological, biochemical, and histological parameters showed no toxicity or tissue damage. In addition, Ruhidas et al. used a double emulsion approach to manufacture etodolac-loaded gum katira microspheres and fed them orally to rats to assess sub-acute toxicity using hematological, biochemical, and histological criteria, and found they were non-toxic [21].

## 6. Methods Used to Modify Gum Katira

Because of the low cost and abundant availability of natural polysaccharides, research on the substitution of petroleum-based polymers with novel natural polysaccharides-based products has intensified in recent years [85,86,87]. However, the short half-lives, rapid degradations by bacteria, changes in viscosity during storage, and low swelling ability and solubility in water of these natural polysaccharides polymers have limited their applications. However, derivatization provides a means of addressing these shortcomings. Various researchers have explored ways of improving the solubility and physicochemical properties of natural gum polysaccharides, and a variety of chemical modifications of polymeric backbones have been investigated over the past decades to explore the possibilities for pharmaceutical, industrial, and biomedical applications. Graft copolymerization and cross-linking procedures are two of the most used ways of modifying the characteristics of natural polysaccharides [88,89,90]. In addition, techniques such as free radical initiation, radiation, and enzyme-assisted grafting are commonly used to modify natural polysaccharides by attaching functional groups [91,92]. Carboxymethylation, sulfation, and thiolation are some of the other chemical modifications attempted [93]. Heat-based [94], freeze–thaw cycling [95], extrusion [96], and micro fluidization [97] are some of the physical methods used to modify natural polysaccharides. On the other hand, chemical modifications of natural gums or polysaccharides have certain advantages over physical or genetic modifications as they are more compatible with large-scale operations and are safe for food and pharmaceutical use [88]. Modifications can also increase the functionality of natural gums, e.g., gel formation, texture, processing capabilities, and synergistic interactions with other components [98], and sometimes produce highly specific effects [99].

The large numbers of hydroxyl groups and occasional carbonyl groups of natural gums and polysaccharides can be used to produce derivatives. The reactions used target carbonyl, hydroxyl, amine, and anomeric hydroxyl groups, and degrees of substitution can define the properties of polysaccharides. Furthermore, the synthetic procedures used to make these derivatives may be selective or non-selective. However, complete substitution can result in undesired events, such as oxidation or depolymerization. Chemical modification techniques are chosen based on several factors, such as the feasibility of modifying specific natural gums or polysaccharides and their potential economic applications in the food or pharmaceutical industries [100]. The various modification methods used are described below.

### 6.1. Carboxymethylation

Natural polysaccharides can be etherified by carboxymethylation, which can produce derivatives easily, cheaply, and safely [101], and this influences the physicochemical properties of gums, such as their solubilities, swelling characteristics, and rheological behaviors, and their powder characteristics [93]. Several natural carboxymethylated polysaccharides have been produced, for example, from starch [102,103], locust bean gum [104], xanthan gum as a release modifier [83], gellan gum as a mucoadhesive polymer [105], gum katira derivatives for nanoparticle production [22], cashew tree exudates [106], guar gum derivatives for textile printing [107], and cellulose [108]. Carboxymethylated gums are more vulnerable to ionic cross-linking, which is attributed to their anionic natures [105]. Carboxymethylation reduced the viscosity of Terminalia gum, enhanced its heat stability, and improved its gelling characteristics [93], and in general, makes polysaccharide gums more anionic. Furthermore, carboxymethylation makes gums more hydrophilic and water soluble and reduces gum viscosities by increasing coulombic repulsion between polymer chains [22], and the introduced carboxyl moiety can be used to synthesize other derivatives via quaternization.

Chloroacetic acid (or its sodium salt) in the presence of sodium hydroxide is commonly used to carboxymethylate polysaccharide gums [100], and degrees of substitution can be controlled by varying chloroacetic acid to sodium hydroxide ratio. The concentration of sodium hydroxide must be high enough to disrupt the crystal structure and allow the modifying agent to penetrate [109,110]. The chloroacetates is converted to glycolate during the reaction [100]. The acid form of carboxymethylated polysaccharides (natural gum, -O-CH_2_-COOH) is formed when chloroacetic acid is used without sodium hydroxide, and intermolecular lactones can be produced due to free carboxyl/hydroxyl condensation during drying, though this is avoided in the presence of NaOH.

Sodium hydroxide and monochloroacetic acid were used to produce carboxymethylated gum katira [22]. Briefly, gum katira was dispersed in an ice-cold sodium hydroxide solution; monochloroacetic acid (75%, *w*/*v*) was added, and the reaction mixture was heated to 70 °C for 30 min with stirring. The mixture was then chilled, suspended in 80% (*v*/*v*) methanol, and filtered to collect the precipitate. Precipitates were neutralized using glacial acetic acid, washed three times in 80% (*v*/*v*) methanol, filtered, and dried in an oven at 40 °C. The carboxymethylated gum katira produced was odorless and colorless. Carboxymethylation was confirmed by FTIR and XRD, which also confirmed its crystallinity, and thermal investigations confirmed its greater thermal stability. Furthermore, SEM showed carboxymethylation transformed thin flaky, smooth-surfaced particles into polyhedral sharp-edged particles with rough surfaces. The carboxymethylated gum was also used to make nanoparticles using chitosan, which was then tested for the delivery of ocular medication [22].

### 6.2. Graft Polymerization

**Figure 4:**
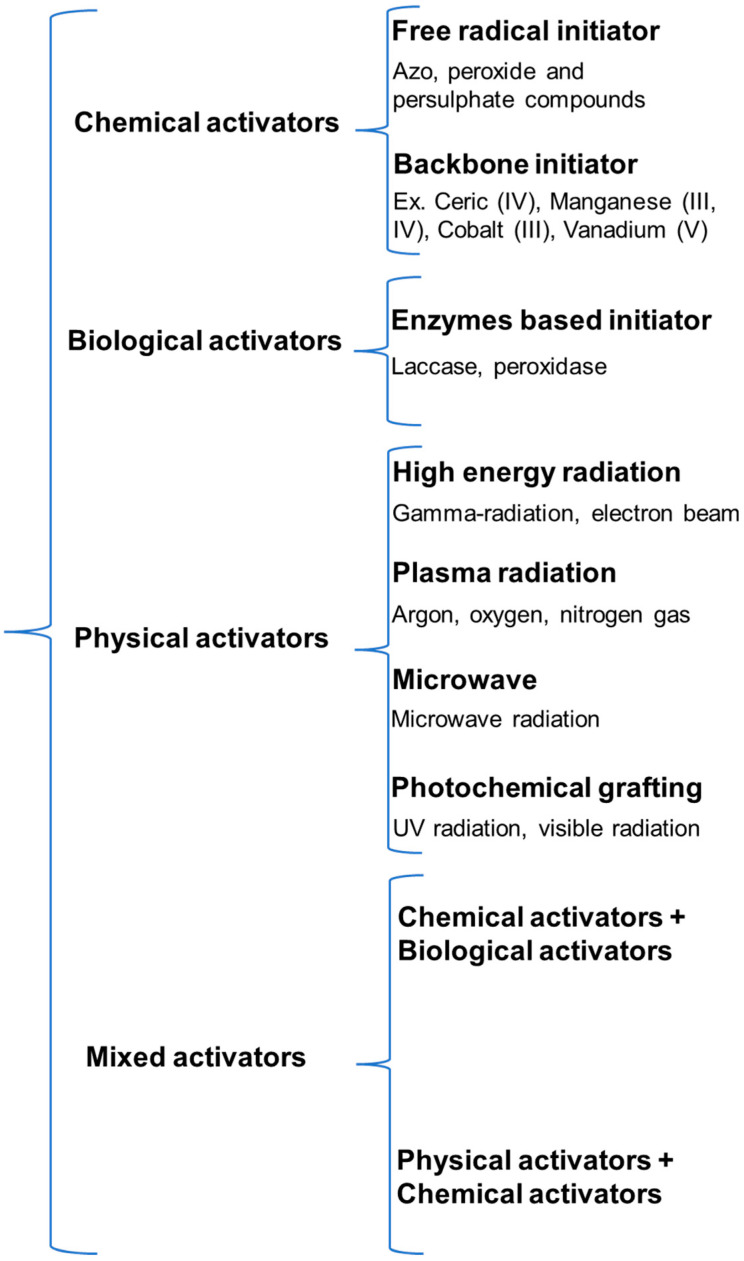
Activator types used for graft polymerization.

Graft copolymers are often composed of large macromolecular weight chains of one monomer (backbone polymer) grafted with other polymers or monomers [86,111,112]. The syntheses of graft copolymers begin with the creation of initiating groups on polymer backbones. To achieve this, pre-synthesized backbone polymers with starter groups connected to the polymer backbone are commonly used. Graft copolymer side chains are typically generated from the polymer backbone via ring opening or free radical initiation polymerization. Free radical graft copolymerization can be performed using hydroxyl groups (-OH) on polymeric chains and redox/thermal initiators such as azobisisobutyronitrile, ammonium persulfate, ferrous ammonium sulfate, thiocarbonate potassium bromate, or potassium diperiodatocuprate (III), or redox initiation pairs such as ascorbic acid/K_2_S_2_O_4_, H_2_O_2_/Fe^2+^, K_2_S_2_O_4_/Fe^2+^, oxalic acid/KMnO_4_, Cu^2+^/Na_2_S_2_O_5_ [113], potassium peroxodisulfate, sodium persulfate, or ceric ammonium nitrate [13,29,114,115]. Figure 4 provides a summary of polymerization activator types.

### 6.3. Synthesis of Grafted and Crosslinked Katira Gum by Free Radical Initiation

A novel hydrogel poly(acrylamide-co-poly-N-methylacrylamide)-grafted gum katira was synthesized by free radical copolymerization using different amounts of acrylamide and N-methylacrylamide [116]. Briefly, gum katira was suspended in doubly distilled water, and then acrylamide, N-methylacryamide, N,N’-methylene-bis-acrylamide (MBA), and potassium peroxodisulfate (KPS; the initiator) were added and heated for 3 h at 70 °C under flowing nitrogen. The reaction was stopped by adding hydroquinone. The hydrogels produced were dried in an oven at 45 °C to constant weight. The formula was optimized based on swelling properties, and the hydrogel produced was evaluated by FTIR, XRD, and SEM and used to remove textile dyes, e.g., methylene blue (MB), malachite green (MG), and Congo red (CR), from single and ternary solutions. The effects of reaction time, pH, and temperature on hydrogel swelling were investigated, and it was found for the optimized product that swelling percent increases with time and reached a maximum at 7 h. On the other hand, the swelling percentage peaked at pH 5.5. The protonation of hydrogel amide groups (-CONH_2_/-CONHMe) caused repulsion between polymeric chains and resulted in a swelling shift to lower pH. The swelling percentage also increased with temperature and peaked at 50 °C, after which it fell due to reduced crosslinking and hydrogen bond disruption. This was also associated with a reduction in water content and swelling.

**Figure 5:**
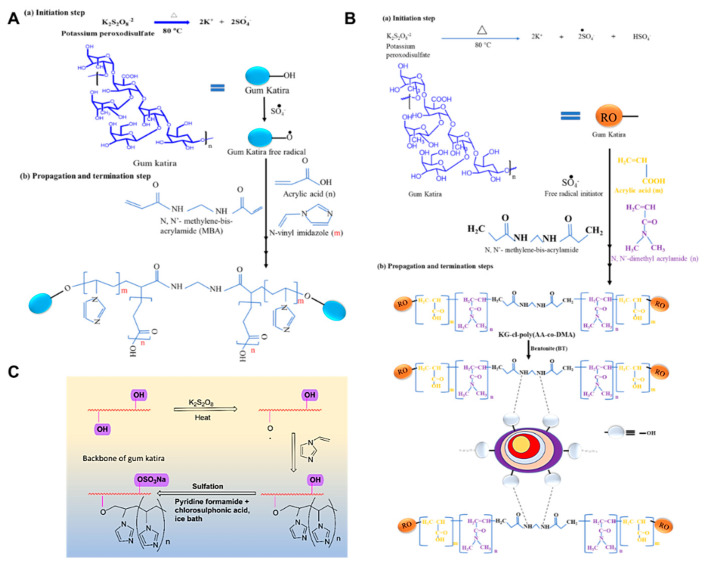
Proposed mechanism for the synthesis of (**A**) gum katira − cl-poly(acrylic acid − co − N − vinyl imidazole) [13] and (**B**) for the synthesis of KG − cl − poly(AA − co − DMA)@BT [116]. (**C**) Schematic of the synthesis of sulfated gum katira using the grafting method. Reprinted/adapted with permission from [25].

Jana et al. synthesized a pH-sensitive gum katira using a free radical method [13]. KPS was used as the initiator, MBA as the crosslinker, and N-vinyl imidazole (NVI) and acrylic acid (AA) as monomers. Dissolved oxygen in the reaction mixture was removed by nitrogen purging. The polymerization was carried out at 80 °C for 3 h, and acetone was used to remove unreacted initiator and monomers. The final product was dried in a hot oven at 50 °C for 24 h. Grafting efficiency was improved because the potassium persulfate formed a sulfate anion free radical in this reaction. This radical anion is formed when KPS is thermally decomposed and abstracts protons from the hydroxyl groups of the gum katira backbone to produce radicals. In the presence of MBA, these radicals reacted with NVI and AA to propagate polymer chains and produce a 3D cross-linked structure. FTIR, SEM, and XRD studies were performed on the pH-sensitive polymer produced, and swelling experiments were carried out at different pH values. The swollen hydrogel (poly-ampholytic network) exhibited the greatest absorptions at pH 4.5 and 9. A proposed mechanism for the synthesis of gum katira-cl-poly(acrylic acid-co-N-vinyl imidazole) is provided in Figure 5A.

A pH-sensitive MBA crosslinked nanocomposite was produced using an in situ copolymerization technique by reacting gum katira with N, N-dimethylacrylamide (DMA), and AA (monomers), bentonite, and KPS (the initiator) [15]. Bentonite is an aluminum phyllosilicate with a high surface area, cation exchange ability, hydroxyl groups, and a swelling property, all of which increase its ability to absorb anionic, cationic, and organic pollutants [117,118]. In brief, gum katira was dispersed in distilled water and stirred at 300 rpm at 70 °C for 30 min. AA and MDA were then added, followed by MBA with vigorous stirring. The reaction was conducted under N_2_ to remove dissolved oxygen. KPS was then added, and the mixture was continuously agitated for 3 h at 70 °C and allowed the stand at room temperature for 24 h. The gel was precipitated by adding acetone, washed with alcohol, and dried at 55 °C.

A gum katira-cl-poly(acrylic acid-co-N, N-dimethylacrylamide)/bentonite nanocomposite hydrogel was synthesized in a similar manner (Figure 5B) [15]. To produce a homogeneous dispersion, bentonite was dispersed in doubly distilled water and sonicated for 30 min. Then, under continuous stirring at 300 rpm, gum katira was added to the reaction mixture, followed by AA, DMA, and MBA. The dissolved oxygen was removed by passing N_2_ through the reaction mixture. KPS was then added, and the mixture was heated at 80 °C with continuous stirring for 3 h and then allowed to stand at room temperature for 12 h. The gum was then precipitated with acetone, washed with a 5:1 methanol/water mix, and dried for 24 h at 50 °C. The nanocomposite hydrogel produced had a significantly higher swelling percentage than gum katira and its water swelling behavior was pH dependent and peaked at pH 10.

Kolya et al. reported a grafted gum katira hydrogel based on poly (N-vinyl imidazole) [25]. Briefly, poly (N-vinylimidazole) monomer and potassium persulfate were used to produce the hydrogel. Chlorosulfonic acid, in the presence of anhydrous pyridine and formaldehyde, was used to sulfate the gum katira graft copolymer produced (Wang et al., 2013). The polymer was characterized by FTIR and FESEM. The synthesis of the grafted hydrogel was confirmed by morphological changes. Under certain conditions, the sulfated graft copolymer absorbed more water than the graft copolymer. A schematic of the grafting technique used is provided in Figure 5C.

**Figure 6:**
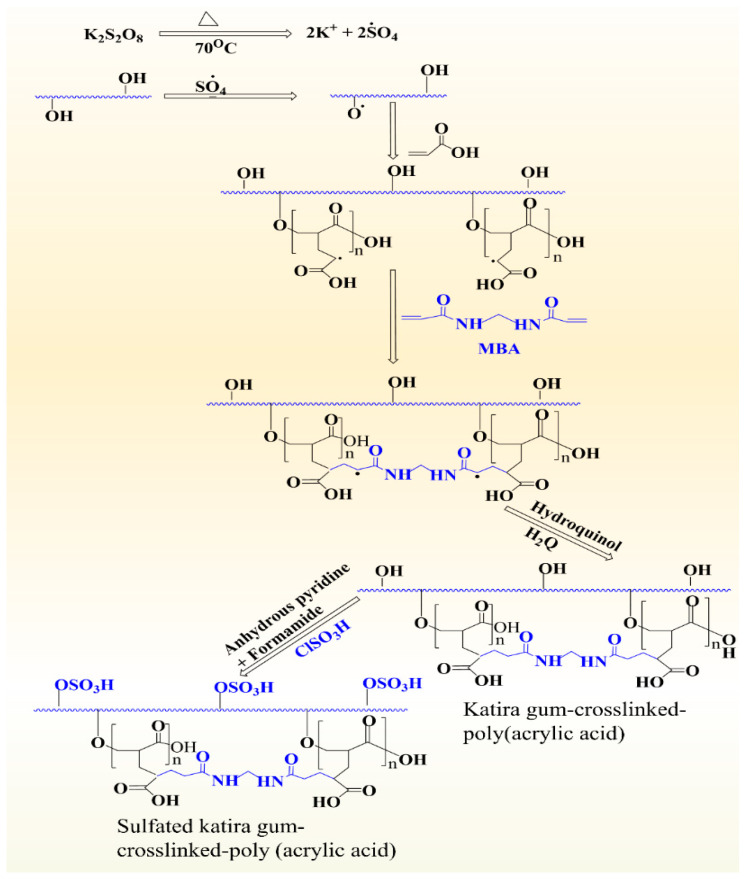
Sulfation of gum katira-crosslinked poly(acrylic acid) hydrogel using chlorosulfonic acid in the presence of anhydrous pyridine and formamide to produce sulfated gum katira-crosslinked poly(acrylic acid). Reprinted/adapted with permission from [15].

Jana et al. manufactured a highly anionic-sulfated gum katira-crosslinked poly(acrylic acid) hydrogel to remove vanadium from wastewater (Figure 6) [15]. The hydrogel was produced by free radical polymerization using KPS as the free radical generator and MBA as the crosslinker. Briefly, gum katira was dispersed in doubly distilled water in a conical flask for 30 min, and then MBA and acrylic acid were added. The reaction mixture was then homogenized for 1 h under N_2_. KPS was added, and the mixture was heated for 3 h at 70 °C. The reaction was then stopped using hydroquinone solution, and the reaction mixture was cooled to room temperature, added to a 75:25 methanol–water mixture, and left overnight. The product was rinsed in excess acetone and dried up at 60 °C in a vacuum oven to consistent weight. The sulfation of the product was accomplished by adding chlorosulfonic acid in pyridine dropwise to a suspension of the gum katira crosslinked poly(acrylic acid) in formamide in a double-necked Erlenmeyer flask at 30 °C with continuous stirring. The reaction mixture was then heated for 3 h at 60 °C, cooled, and poured into an aqueous ethanol solution of pH 8. The hydrogel produced was collected, dried, and subjected to FTIR, XRD, and SEM. Swelling properties were investigated using equilibrium swelling ratio (ESR) at 70 °C and pH 8.5. The sulfated gum katira crosslinked poly(acrylic acid) had an ESR of 2376, whereas the gum katira-crosslinked poly(acrylic acid) had an ESR of 2241.

The influence of contact time, pH, temperature, and salts on percent ESR were also examined. The swelling percentage of hydrogels increased with time and reached a maximum after 11 h, and the hydrogel swelled more in basic media. Hydrogel swelling increased with temperature, peaked at 70 °C, and then declined due to intramolecular hydrogen bond disruption and cleavage of the polymeric backbone. Furthermore, the swelling capacity was dramatically reduced in the presence of an inert salt. Enzyme-assisted graft polymerization can be used to modify gum katira and adapt its properties to meet specific needs. Enzyme-assisted grafting contributes significantly to the characteristics of gum katira, such as chirality and biodegradability. Furthermore, chemo-, stereo-, or position-selective enzyme-based catalysis can be used with other techniques to create novel polymers [119,120].

## 7. Applications of Gum Katira Polysaccharide

Natural polymers are being increasingly used in various fields, such as for drug delivery, because they are readily available, inexpensive, stable, non-toxic, and biodegradable. Polymeric delivery systems are used to achieve the desired pharmacological effects in animals and humans. Drug concentrations at targeted sites must be in therapeutic ranges but are often difficult to achieve for a variety of reasons, such as in vivo degradation, difficulties reaching targeted sites, interaction with non-targeted cells, and bioaccumulation. To address these issues, polymeric delivery systems must be specifically adapted to meet requirements. Natural polymers appear to be a feasible option for developing drug delivery systems and offer the advantages of large-scale production, cost-effectiveness, and patent compliance [121]. 

**Figure 7:**
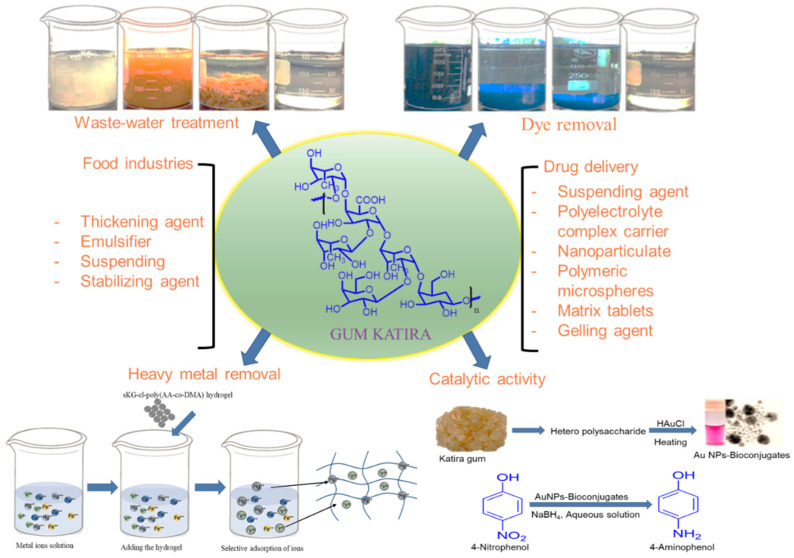
Applications of gum katira and modified gum katira.

The presence of hydroxyl groups and anionic charges in gum katira increases cell membrane permeability, which promotes active molecule absorption and bioavailability [49]. Because of these characteristics, gum katira polysaccharide is frequently employed as a drug delivery vehicle, and its biodegradability and lack of cytotoxicity make it a viable alternative to synthetic polymers for long-term drug administration. Various applications of gum katira and its derivatives are discussed in this section, which includes nanoparticle, hydrogel, matrix, microsphere, suspending and gelling agents, and drug delivery systems. In addition, to drug delivery, the gum has a wide range of uses, such as wastewater treatment, dye removal, and heavy metal adsorption (Figure 7).

### 7.1. Gum Katira in Matrix Tablets

Matrix tablets are designed to release drugs over extended periods. Release modifiers, which are excipients matrix tablets, provide this extended-release effect [122]. Several synthetic polymers can be used as release modifiers for extended-release tablets, and pharmaceutical scientists are actively investigating the use of natural polymers as release modifiers due to their numerous advantages over semi-synthetic and synthetic polymers [122,123,124,125,126]. The abilities of gums from various plants to act as release modifiers for the manufacture of matrix tablets have been well reported [127,128,129]. Using tramadol as a model drug, Singh et al. developed matrix tablets using gum katira as a release retardant [130]. Polymeric hydration and relaxation were found to be involved in drug release from gum katira-containing tablets. Bharaniraja et al. investigated the usages of katira gum, hydrogel, and grafted gum as carriers for colon-specific ibuprofen delivery using in vitro techniques [28]. Gum katira at 10–30% in formulation prevented the drug from being completely released in the stomach and small intestine and performed better than grafted gum and hydrogel. Girotra and Singh produced matrix tablets of gum katira for the delivery of azathioprine to the colon [51], and almost all of the remaining drug was released in the colon. The release mechanism was determined to involve a combination of diffusion and erosion.

### 7.2. Gum Katira as a Gel

Diverse gum katira and derivative hydrogels have been designated for various applications. Girotra and Singh used a combination of gum katira (3.75%) and silver sulphadiazine (1%) and found a substantial increase in burn wound contraction and reduction in epithelialization time for this combination as compared with gum katira or silver sulphadiazine alone [131]. Furthermore, using a free radical initiation approach, Kolya et al. produced sulfated gum katira-g-Poly (N-vinyl imidazole) hydrogels to scavenge mercury (II) ions from aqueous media [25]. A pH-responsive hydrogel was synthesized by grafting a combination of acrylic acid and N-vinyl imidazole onto gum katira using a free radical co-polymerization process, and this gel was then used to remove anionic and cationic dyes from water [13]. In addition, Jana et al. created a poly (acrylic acid) grafted gum katira hydrogel and used it to remove vanadium (IV) ions from water [15].

### 7.3. Gum Katira-Based Polymeric Microspheres

Microparticulate-based drug delivery for continuous release administration has been well investigated [132], and polymeric microspheres have been used to deliver drugs in a targeted and rate-controlled manner. Drugs are released from microspheres by polymer matrix degradation or leaching, and biodegradable polymers have been reported to be excellent candidates for long-term medication delivery [133]. Ruhidas et al. examined the biochemical and anti-inflammatory properties of etodolac polymeric microspheres made with gum katira [61] and concluded that 1% *w*/*v* gum katira could be used to deliver drugs in a continuous, controlled manner. Furthermore, a biochemical investigation revealed that the use of gum katira resulted in a significant reduction in ulcerogenicity as compared with etodolac.

Site-specific drug delivery provides a means of markedly improving treatment efficacy by reducing undesirable off-target effects [134,135]. Colon-targeting 5-Fluorouracil (5-FU)-loaded gum katira microspheres were produced and demonstrated effective cytotoxicity against the HCT-116 cell line and a high entrapment efficiency and a consistent drug release pattern over 12 h [20]. It was concluded that an optimized polymeric gum katira microsphere/5-FU formulation offered a more effective means of delivering drugs for the treatment of colon cancer.

### 7.4. Gum Katira as Suspending Agent

Pharmaceutical suspensions consist of two phases: a continuous (liquid or semisolid phase) and a dispersed phase (solid phase) [136]. These are thermodynamically unstable mixtures that require a suspending or stabilizing agent to reduce settling rates, allow the redispersion of settled particles, and improve the consistencies of suspensions. Natural polymers (tragacanth, acacia, and xanthan), cellulose derivatives (carboxy methylcellulose and methylcellulose), synthetic polymers (carbomer and polyvinylpyrrolidone), and particulate colloids (veegum and bentonite) have all been used as suspending agents because they produce colloidal gels in water. Gum katira is a semi-transparent gum that is insoluble but forms a hydrogel-like mass in water. Singh et al. compared the suspending abilities of gum katira and acacia gum at concentrations of 1–5% for the manufacture of nimesulide suspensions [137], and demonstrated gum katira was the better suspending agent using a series of experiments and evaluations. Gum katira could be used to manufacture suspension preparations.

### 7.5. Derivatized Gum Katira–Polyelectrolyte Complex-Based Nanoparticles

Polyelectrolytes are polymers with large numbers of ionizable functional units and, in an ionized state, can create polyelectrolyte complexes in solution due to electrostatic interactions between oppositely charged polyelectrolytes [138,139,140]. Polymer structures, positions, and natures of ionic groups, charge propagation through polymeric chains, polymer chain flexibility, and pH and temperature of reaction media influence the formation of polyelectrolyte complexes [139]. Several investigators have examined the possibility of using polyelectrolyte complexes as drug delivery systems [141,142,143]. Carboxymethylated gum katira has been tested as a drug delivery system [22]. Polyelectrolyte complex nanoparticles were optimized utilizing a two-factor and three-level central composite design for the ocular administration of ofloxacin. The best formula had a particle size of 269 nm, an entrapment effectiveness of 83.65%, and a drug release rate of 92% after 24 h, as determined by Higuchi release kinetics with Super case-II. The optimized formula exhibited enhanced drug permeability against an aqueous solution through isolated porcine corneas. Similarly, using ofloxacin as a model drug, polyelectrolyte complexes of carboxymethyl gum katira (CMGK) and chitosan (CH) were synthesized and assessed for drug delivery [144]. The best polymer ratio (CMGK/CH) for a drug loading of 50% *w*/*w* was 2.13. The optimized CMGK/CH polyelectrolyte complex formula had a yield of 69.04% and an entrapment efficiency of 84.86% for ofloxacin. Furthermore, Higuchi’s square root release kinetics for the optimized CMGK/CH polyelectrolyte complex formula showed a drug release of 84.32% in 24 h.

In another study, Bernela et al. synthesized glycyrrhizic acid-loaded nanoparticles of poly(cationic) chitosan or poly(anionic) gum katira and tested their anti-inflammatory effects in a carrageenan rat hind paw inflammation model [23]. The anti-inflammatory action of glycyrrhizic acid was enhanced when it was encapsulated in chitosan–gum katira nanoparticles. By using a three-factor, three-level central composite experimental design, the different abilities of glycyrrhizic acid, chitosan, and gum katira were studied for encapsulation effectiveness and glycyrrhizic acid particle size. The optimized nanoparticulate formulation had an encapsulation efficiency of 84.77% and a particle size of 175.8 nm.

### 7.6. Gum Katira in Ayurvedic Medicine

Because of its therapeutic characteristics, gum katira plays an important part in ayurvedic medicine and has been used to treat many ailments, such as coughs and dysentery, since ancient times [24]. After soaking in water overnight, gum katira transforms from a crystalline state to a white jelly, which can be used to cool the body in summer and heat the body in winter. The gum katira has a purgative effect, as it accelerates the peristalsis in the gastrointestinal tract, and it also promotes regular bowel movements and digestive tract health. It is also used to treat acne, pigmentation, dryness, and a variety of skin issues. Due to its cooling properties, the gel form is administered to breast-feeding animals in the summer to increase milk supply. It is also used as an immunity booster to increase immune cell release and strengthen the body’s defenses against pathogens.

### 7.7. Gum Katira Acts as a Catalyst for Nanoparticle Synthesis

Gum katira has also been used to produce gold nanoparticles using a green synthetic technique [145]. During the process, gum katira also functions as a stabilizing and reducing agent. Gold nanoparticles exhibit considerable stability and do not agglomerate even after three months of storage at ambient temperature. The catalytic activity of gum katira was demonstrated for the reduction of 4-nitrophenol in the aqueous phase to 4-aminophenol; the reaction proceeded with pseudo-first-order kinetics.

### 7.8. Gum Katira in Tissue Culture

In vitro root and shoot production by *Syzygium cuminii* and somatic embryogenesis in Albizia lebbeck have both been successfully achieved using gum katira as a tissue culture gel medium [146]. This medium had a viscosity of less than one-sixth of that of agar media. To improve the firmness of the medium, several combinations of agar (0.2–0.6%) and gum katira (3%) were used. It was concluded that gum katira could be used as a plant tissue gelling agent and is an effective and cost-effective culture medium.

## 8. Impact of Modified Gum Katira on Toxic Dye Removal from Wastewater

### 8.1. Removal or Adsorption of Heavy Metals

In wastewater, nickel, copper, mercury, iron, and vanadium ions present serious health hazards. Several procedures have been developed to remove heavy metal ions, such as neutralization, precipitation, ion exchange, and adsorption [147]. Activated carbon is also used to remove metals from industrial waste, but is expensive, which limits its use. A variety of low-cost natural polymers have been used to remove heavy metals from waste water [148], and gum katira and its derivatives have been shown to have considerable potential for the removal of heavy metal ions from water [25,26] (Figure 7).

**Figure 8:**
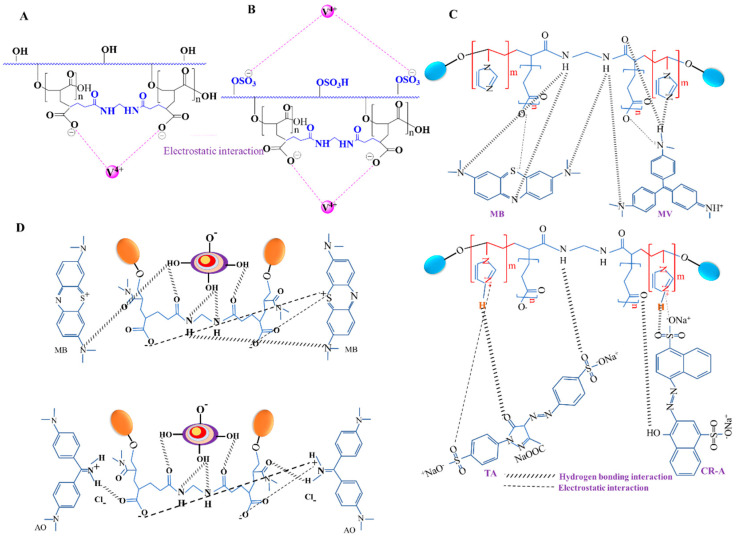
Suggested mechanisms for the interactions between the vanadium (IV) ion and (**A**) gum katira-crosslinked poly(acrylic acid), and (**B**) sulfated gum katira −crosslinked poly (acrylic acid) hydrogel. Reprinted/adapted with permission from [15]. (**C**) Possible absorption mechanism for cationic and anionic dye removal from aqueous solutions. Reprinted/adapted with permission from [13]. (**D**) Putative mechanism of dye removal from aqueous solutions. Reprinted/adapted with permission from [26].

Sulfated gum katira hydrogel was used by Kolya et al. to scavenge mercury (II) ions from an aqueous solution [25]. The coulombic interaction between the Hg (II) ions and the -SO_3_ group contained in the sulfated graft copolymer was shown to underlie the mechanism of adsorption. The sulfated graft copolymer was found to absorb Hg (II) better than the graft copolymer. Sulfated gum katira grafted with poly (acrylic acid) in the form of a hydrogel has also been used to remove vanadium (IV) ions from aqueous solutions [15]. Negative Gibbs free energy and positive entropy values supported the spontaneous absorption of vanadium (IV) by the sulfated hydrogel. Accordingly, sulfated gum katira grafted with poly (acrylic acid) hydrogel can be employed as an adsorbent for removing vanadium (IV) ions from contaminated wastewater. The suggested interactions between the vanadium (IV) ion and gum katira-crosslinked-poly(acrylic acid) and sulfated gum katira-crosslinked-poly (acrylic acid) hydrogels are shown in Figure 8A,B.

### 8.2. Removal of Dyes

Before dyes can be discharged into the environment, they must be removed from industrial effluents and wastewater. To remove different dyes from wastewater, a variety of management strategies have been developed, such as coagulation/flocculation [149], membrane separation [150], electrochemical [151], activated carbon adsorption [152], chemical precipitation [153], and other adsorption techniques [154]. However, the majority of these procedures have high operational costs and are relatively non-selective. Because of its simplicity, cost-effectiveness, efficiency, and ability to recycle adsorbents, adsorption is the most valuable treatment technique.

Polysaccharide preparations are currently being used as adsorbents to remove dyes from water. Jana et al. made a pH-responsive hydrogel by grafting a combination of N-vinyl imidazole and acrylic acid onto gum katira using a free radical co-polymerization process and N, N’-methylene-bis-acrylamide as the cross-linking agent and KPS as the initiator [13]. The developed hydrogel was used to remove cationic (methyl violet (MV) and methylene blue (MB)) and anionic (Carmoisine-A (CR-A) and Tartrazine (TA)) dyes from aqueous solutions (Figure 8C). Anionic dyes were adsorbed at pH = 2 for TA and pH = 3 for CR-A, while cationic dyes were adsorbed at pH = 7. As a result of this pH dependency, adsorptions followed pseudo-second-order kinetics, according to the Langmuir isotherm model. For MB, MV, CR-A, and TA dyes, the hydrogel had maximal adsorption capacities of 331.5, 286.01, 273.5, and 201.53 mg/g, respectively. Dye adsorption by the hydrogel was mediated by electrostatic and hydrogen bond interactions due to the presence of acidic and basic functional groups, as illustrated in Figure 7 and Figure 8. Since interactions of cationic dyes had greater positive entropy values than anionic dyes, they were adsorbed more strongly. Furthermore, positive interaction enthalpies indicated that adsorption processes are favorable at higher temperatures.

Gum katira and its modified acrylic acid and N, N-dimethylacrylamide form mixed with bentonite were utilized in another investigation to remove cationic dyes such as Crystal violet (CV), Methylene blue (MB), and Auramine-O (AO) from aqueous solutions (Figure 8D) [15,26]. Medium pH altered cationic dye adsorption, and maximal dye adsorptions occurred at different pHs for different cationic dyes. Maximum adsorption occurred at pH = 8 (86.87%) for MB, pH = 7.5 (84.29%) for CV, and pH = 10 (83.93%) for AO, while the removal effectiveness fell as pH increased. At 0.11 g of adsorbent, the maximum removal efficiencies were 93.1%, 91.73%, and 90.9% for MB, CV, and AO, respectively. These results can be attributed to a concentration-dependent increase in the number of adsorption sites. Additionally, because ionic interactions were governed by the surface charges of the adsorbent and adsorbate in an aqueous solution, cationic dyes were adsorbed by the gum katira-cl-poly(acrylic acid-co-N-vinyl imidazole) hydrogel [15,155]. The ability of modified grafted gum katira to remove dyes from aqueous solutions could provide a useful means of wastewater treatment. The adsorptions of dyes and the interactions involved are shown in Figure 8D.

Using MBA as a crosslinking agent, a gum katira-based hydrogel was created by grafting poly (acrylamide-co-N-methylacrylamide) onto its backbone [13,15]. The synthesized hydrogel successfully removed textile dyes, such as MB, MG, and CR, from single and ternary solutions. Thermodynamic tests showed that the adsorptions are spontaneous and exothermic. The order of dye adsorption by the gum katira hydrogel was MB > MG > CR. Several important uses of modified gum katira show that this grafted form of the hydrogel promotes dye removal from wastewater. Applications of gum katira and its modifications are summarized in Table 2.

## 9. Conclusions and Future Scope for the Development of Gum Katira Polysaccharide

Natural gums are attractive propositions for pharmaceutical and other applications because they are readily available, inexpensive, non-toxic, chemically modifiable, biodegradable, and biocompatible with few exceptions. The anionic, hydrophilic, and acidic properties of gum katira and its modifications have wide-ranging applications, for example, as drug delivery, gelling, and suspending agents, and as food ingredients. Chemically, the carboxylic and hydroxyl groups of gum katira can be easily crosslinked or grafted with monomers or other polymer matrices to develop in a variety of industrial applications. However, enzymatic gum katira modification has yet to be investigated. Moreover, there is a need to investigate the potential of synthetic modifications of gum katira to expand its industrial applications. This might be achieved by: (1) Enzymatic modification to increase selectivity and increase the percentage yields and purities; (2) Modifying the hydroxyl group of gum katira polysaccharides with monomers, such as vinyl acetate, 1,2-epoxy-5-hexene, benzyl acrylate, or vinyl cinnamate, for applications in the food and pharmaceutical industries; (3) Producing semi-interpenetrating networks or interpenetrating polymer networks containing gum katira and dissimilar hydrophobic or hydrophilic polymers to advance drug delivery applications; (4) Producing binary graft gum katira derivatives to enhance swelling and stability and the adsorption of dyes and heavy metals from wastewater; and (5) Assessing the influence of crosslinked or grafted gum katira at the gene level to discover its molecular effects on cells. Furthermore, gum katira and its derivatives are non-toxic, and thus, studies are required to investigate its utility in the food industry and investigate its potential biomedical applications, especially for drug delivery. Furthermore, the acidic/anionic nature of gum katira and its derivatives forms might be useful for preventing bacterial biofilm growth. We believe that such studies would result in applications in a variety of fields and that natural gums and their derivatives will provide superior materials for drug delivery, biomedical applications, and the food and textile industries.

## Figures and Tables

**Table 1:** Summary of the physicochemical parameters of gum katira.

Physicochemical Properties	Remarks	References
Molecular Weight	Determined by gel-chromatography and found approx. 1.56 × 105 Da.	[60]
Optical rotation	The gum katira showed a specific rotation of ${\left[\alpha \right]}_{D}^{28.2}$ + 44.8	
Solubility	Slightly soluble in water form reddish brown color hydro-dispersion. Practically insoluble in ethanol, acetone, chloroform, and other organic solvents.	[17,20,72]
Flow property	Good flow properties with Angle of repose 32.32 ± 1.16 and Hausner’s ratio 1.59 ± 0.25, compressibility index: 13.10 ± 0.27	
Moisture sorption capacity and moisture content (%*w*/*w*)	Moisture sorption capacity: 3.5 ± 1.12; Moisture content: 15.63 ± 0.37	
Hydration capacity and Water retention capacity (%*w*/*w*)	The hydration capacity of gum suggests that it is efficient at absorbing about five folds of water by its own weight. The water retention capacity is 21.62 ± 0.50	
pH	The gum possesses acidic nature. The pH of the 1% (*w*/*v*) solution was found to be 5.53 ± 0.25.	
Melting Point	Charring started at a temperature of about 50 °C	
Particle size distribution	A uniform frequency distribution of particles was observed	
Swelling index and swelling capacity	Higher swelling capacity of gum was observed, indicating more water absorbing ability with a swelling index of 4.93 ± 0.15 %*w*/*w*	
Loss on drying (%*w*/*w*)	Found in the limit of 8.266 ± 0.35	
Total Ash (%*w*/*w*)	Low level of contamination was observed due to low total ash value of 0.74 ± 0.026	
Microbial study	Limit confirms the applicability and suitability of gum to be used as excipient in pharmaceuticals	
Volatile acidity (%)	The presence of an acidic component was observed around 5.4 ± 0.32	[60]
Viscosity	Viscosity was found to be reduced upon carboxymethylation	[22]

**Table 2:** Applications of gum katira and its modifications.

Polymer	Drug Used	Type of Formulation	Applications	References
Carboxymethyl gum katira	Ofloxacin	Nanoparticles	Ophthalmic drug delivery	[22]
Gum katira	Tramadol	Matrix tablet	Release modifiers	[130]
Gum katira	5-Fluorouracil	Microspheres	Colon targeted	[20]
Gum katira	Etodolac	Microspheres	Sustained delivery	[61]
Gum katira	Gold	Gold nanoparticles	Reducing and stabilizing agents	[145]
Gum katira	N/A	Hydrocolloids and their blends	Drug delivery	[156]
Gum katira	N/A	Culture media	Gelling agent in tissue culture media	[146]
Chitosan–gum katira	Bromelain	Nanoparticles	Sustain release	[27]
Chitosan–gum katira	Glycyrrhizic acid	Nanoparticles	Sustained release	[23]
Chitosan–gum katira polyelectrolyte complex	Ofloxacin	Polyelectrolyte complex	Sustained release	[144]
Gum katira with acrylamide	Ibuprofen	Matrix tablet	Colon targeted delivery	[29]
Gum katira-crosslinked with poly (acrylic acid-co-N-vinyl imidazole)	N/A	Hydrogel	Adsorption/desorption studies on organic dyes	[13]
Gum katira–poly (acrylic acid-co-N, N-dimethylacrylamide)/Bentonite	N/A	Nanocomposite hydrogel	Removal of cationic dyes	[26]
Gum katira-sulfated graft-poly (N-vinyl imidazole)	N/A	Hydrogel	Mercury ion scavenger	[25]
Sulfated Katira gum–cl-poly (acrylic acid)	N/A	Hydrogel	Removal of vanadium	[26]
Gum kaitra	Silver sulphadiazene	Topical gel	Wound healing	[23]
Gum kaitra	Azathioprine	Matrix tablet	Colon targeted	[27]
Gum katira	Nimuslide	Suspension	Suspending agent	[137]

N/A = Not applicable.
